# The Best of Times and Yet the Worst of Times

**DOI:** 10.7759/cureus.20231

**Published:** 2021-12-07

**Authors:** Emma Greear, Taskeen R Kazmi, Adegbenga Bankole

**Affiliations:** 1 Rheumatology, Carilion Clinic, Roanoke, USA

**Keywords:** systemic lupus erythematosus, child and adolescent, med-peds, inflammatory disease, comorbids of sle, avascular necrosis (avn), steroid adverse effects, pediatric rheumatology, internal medicine and rheumatology

## Abstract

Young adults represent a vulnerable population for multiple reasons. This time in an individual’s life coincides with many personal and professional changes and challenges. Some individuals may embrace the change, but for those with a chronic illness, this transition may be difficult. As one is navigating decisions that will ultimately impact their future, they must also navigate changes related to their healthcare. Here, we discuss a case involving a transition from a pediatric to an adult rheumatology clinic and the impact on the patient. This case will highlight some of the challenges patients face and will explore how this process could be improved for our patients. For many young individuals, this is the best of times as they are transitioning to an adult but also the worst of times as they must now make adult decisions with adult consequences.

## Introduction

The transition from pediatric to adult rheumatology is a time in which patients are at risk for complications. Studies have shown a startling number of pediatric patients may be lost to follow-up, and estimates are as high as 50% [1]. Mitigation strategies have been put in place to help with this transitional period. Guidelines have been created to assist with the transition period. The European Alliance of Associations for Rheumatology (EULAR) and the Pediatric Rheumatology European Society (PReS) released guidelines in 2016 entitled “EULAR/PReS standards and recommendations for the transitional care of young people with juvenile-onset rheumatic diseases [2].” In addition to guidelines, the American College of Rheumatology (ACR) has developed an ACR transition toolkit that is easily accessible on the ACR website. This toolkit aims to provide guidance during this transition period [3]. GotTransition is an organization solely dedicated to helping youth transition to adult healthcare [4]. Despite the existence of guidelines, toolkits, and organizations solely aimed at making this transition seamless, many challenges still exist.

## Case presentation

An 18-year-old African American female presented for transition of care to an adult rheumatology clinic. During her initial visit, she reported pain and locking in her hips, which was aggravated by prolonged walking. Her pain began over one year prior, extended into her knees, and was more severe on the left. The patient had previously completed physical therapy, which did initially help with her symptoms, but her pain had returned and was worsening. No medications helped with the pain. The patient did not have recent trauma, joint swelling, decreased range of motion, weakness, mucosal ulcers, or hair loss. The patient reported a skin rash. Much of this information was provided by the patient’s mother.

The patient’s past medical history included mixed connective tissue disease (MCTD), food and environmental allergies, asthma, anemia, eczema, and prediabetes. She had previously been treated for MCTD with cyclophosphamide, hydroxychloroquine, methotrexate, tocilizumab, rituximab, methylprednisolone, and intravenous immune globulin (IVIG). Her current medications included hydroxychloroquine 200 mg one tablet twice daily, cholecalciferol 125 mcg one tablet daily, famotidine 20 mg one tablet twice daily, cetirizine 10 mg one tablet daily, epinephrine 0.03 mL by intramuscular route as needed for allergic reaction, and albuterol 90 mcg/actuation inhaler two puffs every four hours as needed for shortness of breath. The patient had allergies to shellfish and cephalosporins. Her family history included asthma, hypertension, and diabetes. No one in the patient’s family had an autoimmune disease. She was living at home with her mother, was a high school graduate, and was currently unemployed. She reported no history of tobacco, alcohol, or drug use. The patient was not sexually active.

Vital signs obtained during the initial visit revealed the following: blood pressure, 117/83 mmHg; pulse rate, 77 beats/minute; temperature, 98.4°F; respiratory rate, 19 breaths/minute; height, 5' 6"; weight, 102.4 kg; and body mass index, 36.43 kg/m².

**Figure 1:**
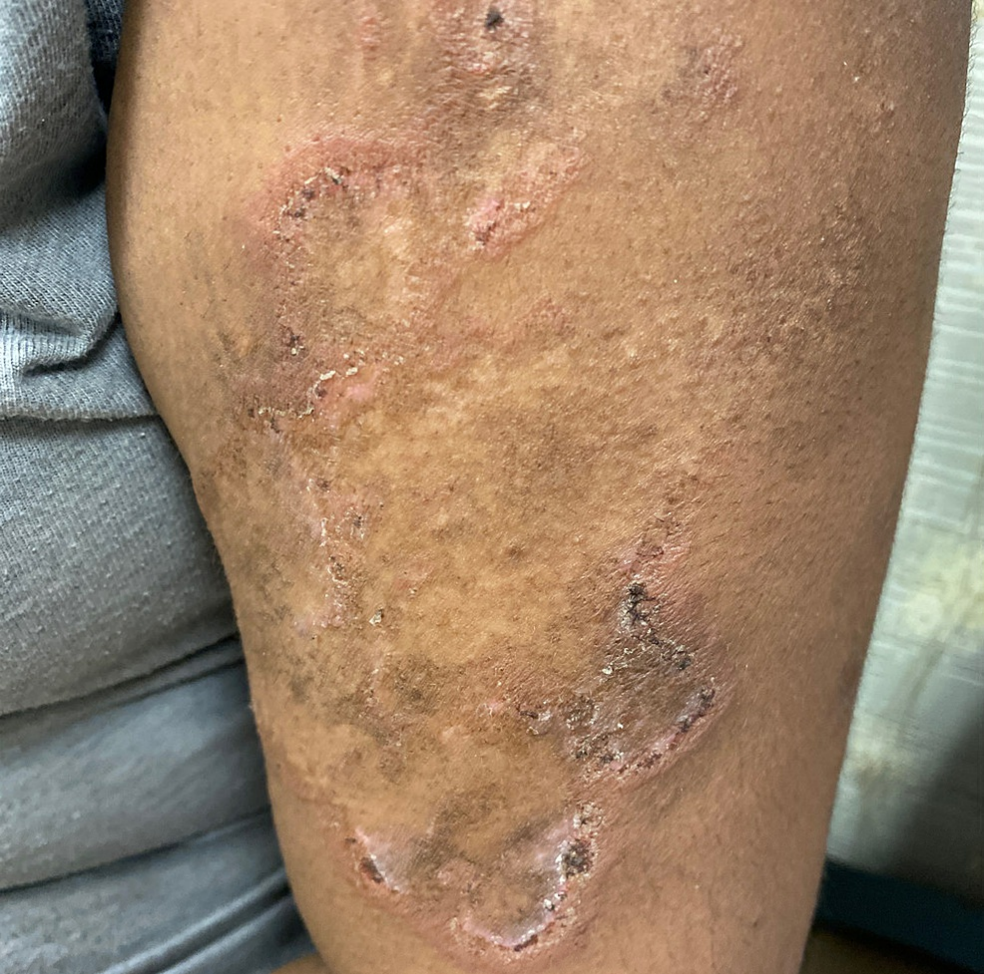
Discoid/annular rash on the upper arm

On physical examination, she was a well-appearing young female in no acute distress. Her skin revealed a discoid/annular rash on her upper right arm (Figure 1). She also had multiple hyperpigmented healing red lesions noted on her anterior chest and arms. No oral or nasal ulcers were noted. Eyes were without redness, and she did not have alopecia or scalp lesions. Joint examination revealed no joint swelling, redness, or warmth. Genu valgum was noted in the right leg and pelvic tilt. The patient’s leg length measurement was as follows: 41 inches from the anterior superior iliac spine (ASIS) to the lateral malleolus bilaterally.

**Figure 2:**
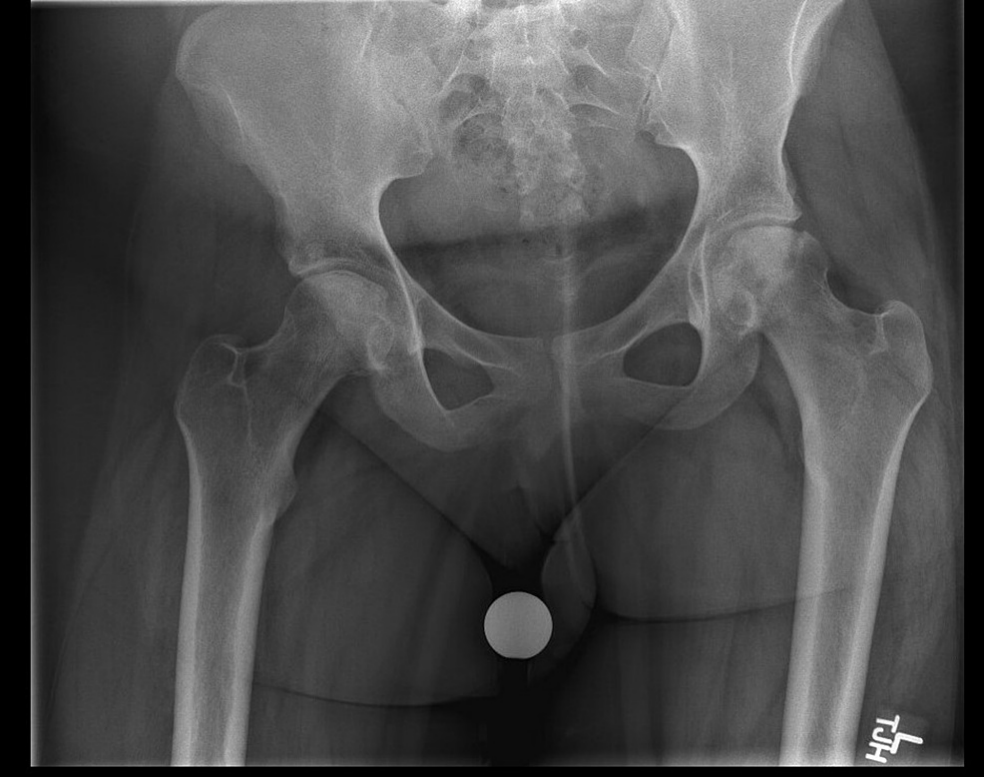
X-ray of the pelvis Single frontal view of the low pelvis demonstrating bilateral femoral head avascular necrosis. Mild subchondral collapse on the left. No definite subchondral collapse on the right.

Imaging revealed bilateral femoral head avascular necrosis (Figure 2). Laboratory studies revealed normocytic anemia, lymphopenia, and positive Smith/RNP antibody (Table 1). Given the findings on imaging, a referral to orthopedic surgery was placed.

**Table 1:** Laboratory studies

Laboratory Study	Reference Range	Result
WBC	4–10.5 K/uL	4.2 K/uL
Hemoglobin	12–16 g/dL	11.7 g/dL
Hematocrit	36%–46%	36.5%
Platelets	130–400 K/uL	306 K/uL
Absolute Lymph	1–5 K/uL	0.7 K/uL
Urea Nitrogen	6–20 mg/dL	11 mg/dL
Creatinine	0.5–1.2 mg/dL	0.50 mg/dL
Alkaline Phosphatase, Serum	47–119 IU/L	74 IU/L
AST	10–42 IU/L	13 IU/L
ALT	10–60 IU/L	11 IU/L
Blood, Urine	Negative	Negative
pH, Urine	5–8	6.5
Protein, Urine	Negative	Trace
RBC, Urine	0–2/HPF	0–2/HPF
WBC, Urine	0–5/HPF	5–10/HPF
Histone Antibody	<1 U	<1 U
ANA Screen, IFA	Negative	Positive
Complement Component C3c	83–193 mg/dL	159 mg/dL
Complement Component C4c	15–57 mg/dL	40 mg/dL
DNA (ds) Antibody	≤4 IU/mL	1 IU/mL
Ribosomal P Antibody	<1 U	<1 U
SM Antibody	<1 U	6.9 U
SM/RNP Antibody	<1 U	>8 U
Sjogren’s Antibody (SSA)	<1 U	<1 U
Sjogren’s Antibody (SSB)	<1 U	<1 U
Thyroid Peroxidase Antibody	<9 IU/mL	<1 IU/mL
SCL-70 Antibody	<1 U	<1 U
Rheumatoid Factor	<14 IU/mL	<14 IU/mL
JO-1 Antibody	<1 U	<1 U
Centromere B Antibody	<1 U	<1 U
Cardiolipin Antibody (IgG)	<20 GPL	<2 GPL
Cardiolipin Antibody (IgM)	<20 MPL	<2 MPL
Cardiolipin Antibody (IgA)	<20 APL	<2 APL
Lupus Anticoagulant	Not Detected	Not Detected
B2 Glycoprotein I Antibody (IgG)	<20 U/mL	<2 U/mL
B2 Glycoprotein I Antibody (IgA)	<20 U/mL	<2 U/mL
B2 Glycoprotein I Antibody (IgM)	<20 U/mL	<2 U/mL
Hydroxychloroquine	100 ng/mL	140 ng/mL
Protein, Urine, Quant	NA	25.9 mg/dL
Creatinine, Urine	NA	352.00 mg/dL
ANA Titer	Negative	≥1:1280
LDH	135–214 IU/L	161 IU/L
CK, Total	26–308 IU/L	113 IU/L
Aldolase	≤8.1 U/L	4.2 U/L
Sedimentation Rate	0–20 mm/hour	22 mm/hour
C-Reactive Protein	<1 mg/dL	<0.40 mg/dL
Complement, Total (CH50)	31–60 U/mL	53 U/mL
C1Q Complement Component	5–8.6 mg/dL	8.5 mg/dL

Orthopedic surgery recommended bilateral total hip replacements due to bilateral avascular necrosis and collapsed femoral heads. Bilateral hip replacements were scheduled, but the patient missed her preoperative appointment. Therefore, her surgical procedure was cancelled. The patient subsequently missed appointments with primary care, cardiology, and rheumatology. A rheumatology follow-up appointment has been rescheduled for the patient.

## Discussion

The period in which patients are expected to transition care coincides with a formidable time in a young person’s life. During this time, young adults are developing autonomy, and a paradigm shift occurs. Decisions that were once left to the parent are now falling on the young adult. During the initial evaluation of this patient, the patient’s mother was very involved and provided much of the history for the patient. Although this practice is common in pediatric practices, adult physicians are not routinely accustomed to obtaining information from parents. Many personal challenges contribute to the overall burden during this transition. Studies show that the following factors are especially impactful: loss or change of insurance, ending long-term relationships with pediatric providers, and difficulty accessing care [5,6].

Although we are aware of the common risks associated with transitioning care from a pediatric to an adult clinic, we must also consider unique challenges that exist in rheumatology. This includes the transition from a pediatric to an adult diagnosis (e.g., from juvenile idiopathic arthritis (JIA) to rheumatoid arthritis (RA)). This calls for a reevaluation of the patient as their disease process is likely still evolving. In addition to reassessing the patient’s diagnosis, we must also consider complications that may have arisen from previous treatments. Patients with a rheumatologic diagnosis have a disease that has systemic manifestations, with this comes its own unique challenges. Many patients are required to follow up with multiple specialists in addition to a rheumatologist, given the systemic nature of their disease. During this period of transition, studies have shown that both morbidity and mortality are increased in young adults. Multiple studies have also shown that patients with pediatric-onset systemic lupus erythematosus (SLE) have increased mortality rates when compared with adult-onset disease [7-9].

As can happen with vulnerable populations, this induvial has missed multiple appointments. This includes appointments with primary care, cardiology, orthopedic surgery, and rheumatology. Could this be due to anxiety about the planned surgical procedure, or does this represent distrust in the medical system? Ultimately, this highlights the need for intensive patient education. A study conducted by Berrios-Rivera et al. looked at trust in physicians in patients with SLE and RA [10]. This study showed lower trust in physicians observed in African American patients. This is consistent with previous studies. The patient discussed in this case is African American; therefore, one must consider that these factors may have contributed to the lack of follow-up [10].

A study conducted by Hersh et al. examined the transition of care from pediatric to adult rheumatology in a US academic center [11]. This study found that the median time between the first visit with an adult rheumatologist and the last visit with a pediatric rheumatologist was greater than seven months. Interestingly, this time delay still occurred in a patient population that was known to have active disease and was transferring care within the same health system [11]. Given the small number of rheumatologists in the United States, especially those that specialize in pediatrics, many patients will not be staying within the same healthcare system, which makes it highly likely that the transition of care may take even longer than what was demonstrated in the study.

## Conclusions

Young adults will be experiencing many life changes during this time of transition. Many of these changes are both exciting and scary, most of which are unrelated to healthcare. Therefore, we must be cognizant of all the varying factors that undoubtedly influence a patient’s overall healthcare. We should also consider the social factors that influence a patient’s decisions and ability to obtain healthcare. In our case, during the patient’s initial visit, her mother was still needed to assist in healthcare-related decisions. It was difficult to determine how much of the disease process and prognosis were understood and accepted by the patient and her family. At her first visit, we did not understand her particular social barriers to care and how this would eventually affect her ability to receive care. This patient could have benefited from the utilization of a transition of care resources such as GotTransition or the ACR transition toolkit. At this time, our patient has been lost to follow-up not only to rheumatology but also to other specialties, as have many other young adults with chronic medical conditions.

This case also forces the medical community to face some questions; should it be a standard of care to have a transitional clinic for these patients? Should such patients be seen by both pediatric and adult rheumatology as part of a formal handoff? Could either of these measures potentially improve outcomes for this subpopulation? Would our patient’s trajectory have changed if her care teams liaised more closely? We recommend that all rheumatology practices should have a plan for the transition of care for young adults, and rheumatology trainees should have exposure to this subpopulation of patients.
